# USP25 inhibition ameliorates Alzheimer’s pathology through the regulation of APP processing and A**β** generation

**DOI:** 10.1172/JCI152170

**Published:** 2022-03-01

**Authors:** Qiuyang Zheng, Beibei Song, Guilin Li, Fang Cai, Meiling Wu, Yingjun Zhao, LuLin Jiang, Tiantian Guo, Mingyu Shen, Huan Hou, Ying Zhou, Yini Zhao, Anjie Di, Lishan Zhang, Fanwei Zeng, Xiu-Fang Zhang, Hong Luo, Xian Zhang, Hongfeng Zhang, Zhiping Zeng, Timothy Y. Huang, Chen Dong, Hong Qing, Yun Zhang, Qing Zhang, Xu Wang, Yili Wu, Huaxi Xu, Weihong Song, Xin Wang

**Affiliations:** 1 State Key Laboratory of Cellular Stress Biology, Fujian Provincial Key Laboratory of Neurodegenerative Disease and Aging Research, Institute of Neuroscience, Department of Neuroscience, Center for Brain Sciences, First Affiliated Hospital of Xiamen University, School of Medicine, Xiamen University, Xiamen, China.; 2 Townsend Family Laboratories, Department of Psychiatry, University of British Columbia, Vancouver, British Columbia, Canada.; 3 Neuroscience Initiative, Sanford Burnham Prebys Medical Discovery Institute, La Jolla, California, USA.; 4 Department of Translational Medicine, School of Medicine, Xiamen University, Xiamen, China.; 5 Department of Pediatrics, Xiang’an Hospital of Xiamen University, Xiamen, China.; 6 School of Pharmaceutical Sciences, Fujian Provincial Key Laboratory of Innovative Drug Target Research, Xiamen University, Xiamen, China.; 7 Institute for Immunology, School of Medicine, Tsinghua University, Beijing, China.; 8 Key Laboratory of Molecular Medicine and Biotherapy, School of Life Science, Beijing Institute of Technology, Beijing, China.; 9 Institute of Aging, Key Laboratory of Alzheimer’s Disease of Zhejiang Province, School of Mental Health and Kangning Hospital, Second Affiliated Hospital and Yuying Children’s Hospital, Wenzhou Medical University, Wenzhou, China.; 10 Oujiang Laboratory, Zhejiang Lab for Regenerative Medicine, Vision and Brain Health, Wenzhou, China.

**Keywords:** Neuroscience, Alzheimer disease

## Abstract

Down syndrome (DS), or trisomy 21, is one of the critical risk factors for early-onset Alzheimer’s disease (AD), implicating key roles for chromosome 21–encoded genes in the pathogenesis of AD. We previously identified a role for the deubiquitinase USP25, encoded on chromosome 21, in regulating microglial homeostasis in the AD brain; however, whether USP25 affects amyloid pathology remains unknown. Here, by crossing 5×FAD AD and Dp16 DS mice, we observed that trisomy 21 exacerbated amyloid pathology in the 5×FAD brain. Moreover, bacterial artificial chromosome (BAC) transgene–mediated USP25 overexpression increased amyloid deposition in the 5×FAD mouse brain, whereas genetic deletion of *Usp25* reduced amyloid deposition. Furthermore, our results demonstrate that USP25 promoted **β** cleavage of APP and A**β** generation by reducing the ubiquitination and lysosomal degradation of both APP and BACE1. Importantly, pharmacological inhibition of USP25 ameliorated amyloid pathology in the 5×FAD mouse brain. In summary, we identified the DS-related gene *USP25* as a critical regulator of AD pathology, and our data suggest that USP25 serves as a potential pharmacological target for AD drug development.

## Introduction

Down syndrome (DS), the most common form of intellectual disability, arises from total or partial triplication of chromosome 21. As one of the critical risk factors for early-onset Alzheimer’s disease (AD), trisomy 21 not only increases amyloid precursor protein (*APP*) gene dosage but also promotes the amyloidogenic pathway in the AD human brain by altering APP processing (1–3) and amyloid plaque deposition in the AD mouse brain (4). In addition to APP and BACE2, it remains to be determined whether other genes on chromosome 21 also contribute to AD pathology in DS (5) and the underlying molecular mechanisms.

By 40 years of age, almost all individuals with DS exhibit AD neuropathology, including amyloid plaques and neurofibrillary tangles (6–9). Moreover, approximately two-thirds of patients with DS develop clinical Alzheimer’s dementia by 60 years of age (10). As the major pathological hallmark of AD, amyloid plaques consist of aggregated amyloid-β (Aβ), a peptide originating from sequential APP proteolysis by β secretase BACE1 and γ secretase (11–13). Cumulative evidence indicates that aggregation of Aβ peptides in vulnerable brain regions is the trigger for tau pathology and gliosis/neuroinflammation. Thus, Aβ targeting may comprise key disease-modifying strategies to treat or prevent AD progression (14–16).

The ubiquitin-specific protease USP25 is encoded by a gene located at 21q11.2. As a negative regulator of IL-17–mediated inflammation, USP25 plays a vital role in innate immunity (17). Previously, we found that USP25 regulates microglial homeostasis and neuroinflammation during neurodegeneration (18). However, it is still unknown whether and how triplication of *USP25* in the DS brain contributes to AD-like neuropathological features, such as amyloid deposition.

Here, we demonstrate that triplication of chromosome 21 genes exacerbated AD-related amyloid pathology in 5×FAD mice. Moreover, overexpression of chromosome 21–encoded *USP25* markedly increased amyloid deposition, whereas *Usp25* deficiency ameliorated amyloid pathology by regulating APP processing and Aβ generation in 5×FAD mice. Furthermore, pharmacological inhibition of USP25 markedly attenuated amyloid plaque burden in 5×FAD mice. Therefore, our results identify a critical role for ubiquitin-proteasome system signaling in the regulation of amyloid deposition and a potentially novel strategy for ameliorating the neuropathology of AD.

## Results

### Increased USP25 gene dosage aggravates amyloid pathology in AD mice.

**Figure 1:**
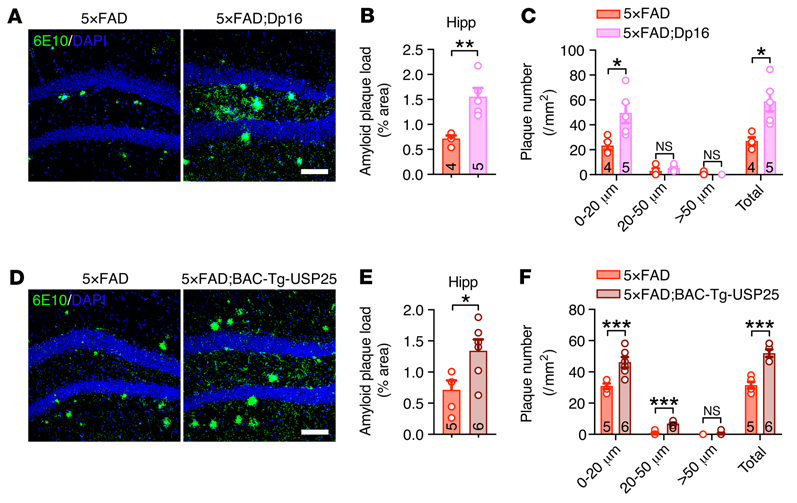
Increased *USP25* dosage promotes amyloid plaque deposition in 5×FAD mice. (**A**–**C**) Representative immunostaining (**A**) and quantification of 6E10-positive amyloid plaques (**B** and **C**) in the hippocampi (Hipp) of 5-month-old 5×FAD (*n* = 4) and 5×FAD;Dp16 (*n* = 5) mice. Scale bar: 100 μm. (**D**–**F**) Representative immunostaining (**D**) and quantification of 6E10-positive amyloid plaques (**E** and **F**) in the hippocampi of 6-month-old 5×FAD (*n* = 5) and 5×FAD;BAC-Tg-USP25 (*n* = 6) mice. Scale bar: 100 μm. All data are presented as mean ± SEM. *P* values were determined by Student’s *t* test. *P < 0.05; **P < 0.01; ***P < 0.001.

To address whether trisomy 21 influences AD pathogenesis, we generated a combined murine DS-AD model by crossing the 5×FAD AD model with the Dp16 DS mouse model, and immunoblot analyses confirmed an upregulation of USP25, APP, and APP-carboxy-terminal fragments (APP-CTFs) in the cortices of 5×FAD; Dp16 mice compared with that of 5×FAD mice (Supplemental Figure 1, A–F; supplemental material available online with this article; https://doi.org/10.1172/JCI152170DS1). We subsequently immunostained hippocampal sections from 5-month-old 5×FAD and 5×FAD;Dp16 mice and found that overdosage of chromosome 21 genes enhanced amyloid plaque deposition in the 5×FAD;Dp16 mouse brain (Figure 1, A–C).

USP25, which resides on chromosome 21, has been previously shown to regulate neuroinflammation in the DS-AD brain (18). To determine whether USP25 affects amyloid pathology in the context of DS-AD, we crossed 5×FAD mice with bacterial artificial chromosome–Tg-USP25 (BAC-Tg-USP25) mice (Supplemental Figure 1, G and H). We performed immunostaining using hippocampal sections from 6-month-old 5×FAD and 5×FAD;BAC-Tg-USP25 mice and observed an increased number of amyloid plaques in the hippocampi of 5×FAD;BAC-Tg-USP25 mice compared with the hippocampi of 5×FAD mice (Figure 1, D–F). This suggests that USP25 upregulation alone can sufficiently aggravate Aβ pathology.

### Genetic deletion of Usp25 ameliorates amyloid pathology in AD mice.

**Figure 2:**
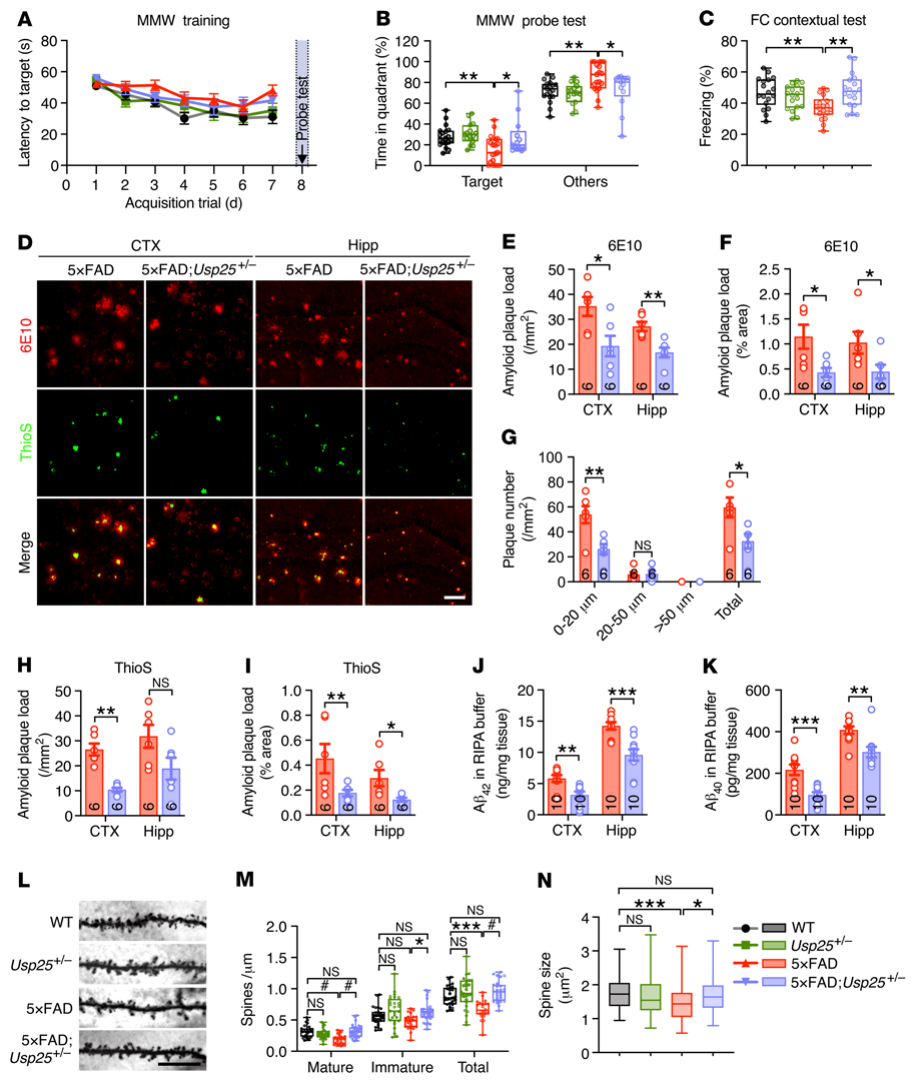
*Usp25* deficiency ameliorates amyloid burden in 5×FAD mice. (**A**) Morris water maze (MWM) test results depicting escape latency, defined as the time taken to find a hidden platform in the 7-day training phase. (**B**) MWM probe test results. *n* = 13–19 mice per group. (**C**) Percentage freezing time in contextual fear conditioning (FC) tests as a readout of associative memory. *n* = 18 mice per group. Six- to 7-month-old mice were used in behavioral tests. (**D**-**I**) Representative immunostaining (**D**) and quantification of amyloid plaques with a 6E10 antibody (**E**–**G**) and thioflavin S (ThioS) (**H** and **I**) in the cortices (CTX) and hippocampi (Hipp) in 6-month-old 5×FAD and 5×FAD;*Usp25^+/–^* mice. *n* = 6 mice per group. Scale bar: 50 μm. (**J** and **K**) Quantification of soluble Aβ_42_ (**J**) and Aβ_40_ (**K**) in RIPA buffer in the cortices and hippocampi of 6-month-old 5×FAD and 5×FAD;*Usp25^+/–^* mice. *n* = 10 mice per group. (**L**–**N**) Golgi staining (**L**) and quantification of mature, immature, and total dendritic spines (**M**) and spine size (**N**) in the cortical layer V regions of 9-month-old WT, *Usp25*^+/–^, 5×FAD, and 5×FAD;*Usp25*^+/–^ mice. Scale bar: 10 μm. *n* = 4 mice per group, 22–27 dendrites per group were counted in **M**, and 95–107 spines per group were counted in **N**. Data are presented as mean ± SEM (**A** and **E**–**K**) or as median with minimum to maximum bars (**B**, **C**, **M**, and **N**). *P* values were determined by 1-way ANOVA with Dunnett’s post hoc analysis in **B** and **C**, by the Mann-Whitney test in **E**–**K**, by 1-way ANOVA with Tukey’s post hoc analysis in **M**, and by Kruskal-Wallis test with Dunn’s post hoc analysis in **N**. *P < 0.05; **P < 0.01; ***P < 0.001; ^#^*P* < 0.0001.

*Usp25*^−/−^ mice displayed normal body weight and cognitive behavior compared with their WT littermates (Supplemental Figure 2); however, it is still unknown whether *Usp25* deficiency affects amyloid pathology. We generated 5×FAD*;Usp25*^+/−^ mice by crossing *Usp25*^+/−^ mice with 5×FAD mice. We evaluated the learning and memory ability of 5×FAD*;Usp25*^+/−^ mice and found that genetic deletion of *Usp25* improved the cognitive performance of 5×FAD mice in the Morris water maze (MWM) test (Figure 2, A and B) and fear conditioning contextual test (Figure 2C). We subsequently performed histological analysis of amyloid plaques in cortical and hippocampal sections from 6-month-old 5×FAD and 5×FAD;*Usp25*^+/−^ mice. Staining with both an Aβ antibody and thioflavin S revealed a reduction in amyloid burden in 5×FAD;*Usp25*^+/−^ mice compared with 5×FAD mice (Figure 2, D–I). We next analyzed the levels of soluble Aβ_40_ and Aβ_42_ in mouse cortical and hippocampal lysates by ELISA and found that genetic deletion of *Usp25* markedly reduced soluble Aβ_40_ and Aβ_42_ levels in the 5×FAD;*Usp25*^+/−^ mouse hippocampus and cortex at 6 months of age (Figure 2, J and K). Furthermore, we performed Golgi staining in cortical sections from 9-month-old WT, *Usp25*^+/–^, 5×FAD, and 5×FAD;*Usp25*^+/−^ mice and observed deficits in dendritic spines in 5×FAD mice and restoration of mature spine density and spine size in 5×FAD;*Usp25*^+/−^ mice compared with 5×FAD mice (Figure 2, L–N). Together, these results demonstrate that *Usp25* downregulation or deletion haploinsufficiency ameliorates amyloid pathology and synaptic deficits in 5×FAD mice.

### USP25 modulates amyloidogenic APP processing in vivo and in vitro.

**Figure 3:**
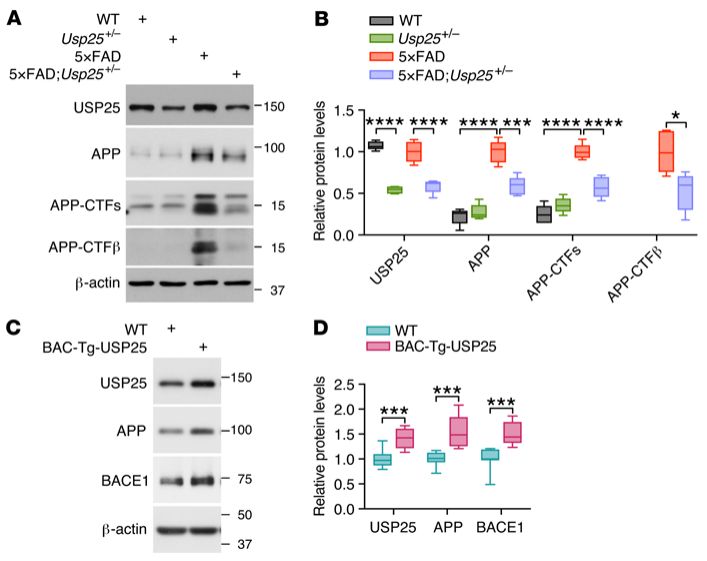
USP25 regulates APP processing in 5×FAD mice. (**A** and **B**) Immunoblot analysis of APP-processing-related proteins in the cortices of 6-month-old WT, *Usp25^+/–^*, 5×FAD, and 5×FAD:*Usp25^+/–^* mice. *n* = 5 per group. (**C** and **D**) Immunoblot analysis of APP, BACE1, and USP25 proteins in the cortices of 6-month-old WT and BAC-Tg-USP25 mice. *n* = 8 per group. The intensity of each immunoblot band was normalized to that of the β-actin band. All data are presented as median with minimum to maximum bars. Values are shown in kDa in **A** and **C**. *P* values were determined by ordinary 1-way ANOVA with Tukey’s post hoc analysis in **B** and by Student’s *t* test in **D**. *P < 0.05; ***P < 0.001; ****P < 0.0001.

The generation of Aβ is derived from sequential cleavage of the transmembrane precursor APP by β and γ secretases (19). To determine whether reduced amyloid deposition with USP25 downregulation is derived from changes in APP processing, we characterized the expression of various proteolytic readouts from APP-processing pathways by immunoblot analysis. We found that *Usp25* deficiency reduced the levels of APP and its cleavage products (both α- and β-CTFs) in the cortices of 6-month-old 5×FAD;*Usp25*^+/−^ mice (Figure 3, A and B), whereas overexpression of USP25 markedly increased the expression of APP and BACE1 in the cortices of 6-month-old BAC-Tg-USP25 mice (Figure 3, C and D).

**Figure 4:**
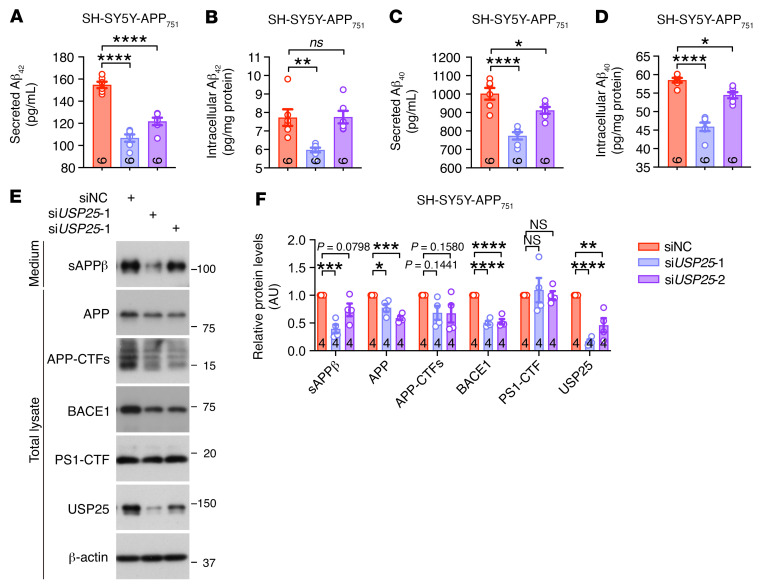
Knockdown of *USP25* reduces APP processing and Aβ generation in vitro. (**A**–**D**) Quantification of secreted and intracellular Aβ_42_/Aβ_40_ in SH-SY5Y-APP_751_ cells upon *USP25* siRNA treatment. *n* = 6 per group. (**E** and **F**) Immunoblot analysis of APP-processing-related proteins in SH-SY5Y-APP_751_ cells upon *USP25* siRNA treatment. The intensity of each immunoblot band was normalized to that of the β-actin band. Values are shown in kDa in **E**. *n* = 4 per group. All data are presented as mean ± SEM. *P* values were determined by ordinary 1-way ANOVA with Dunnett’s post hoc analysis. *P < 0.05; **P < 0.01; ***P < 0.001; ****P < 0.0001.

Consistent with these results in vivo, we found that *USP25* knockdown markedly reduced the levels of secreted and intracellular Aβ_40_ and Aβ_42_ in the human APP-overexpressing SH-SY5Y-APP_751_ cell line (Figure 4, A–D). Moreover, knockdown of *USP25* reduced the expression of APP, APP-CTFs, sAPPβ, and BACE1 but not PS1-CTF (Figure 4, E and F). In addition, overexpression of USP25 increased APP protein levels in APP-overexpressing HEK293T cells (Supplemental Figure 3, A and B). Inhibition of lysosomal degradation by leupeptin abolished the effects of USP25 overexpression on APP, indicating that USP25 affects APP turnover (Supplemental Figure 3, A and B). We also found that overexpression of USP25 increased BACE1 expression, while overexpression of ubiquitin decreased BACE1 protein levels (Supplemental Figure 3, C and D), suggesting that USP25 regulates BACE1 expression through BACE1 ubiquitination. However, downregulation of *USP25* failed to affect the expression of chromosome 21–encoded BACE2 (Supplemental Figure 3, E and F). These results demonstrate that USP25 enhances Aβ accumulation through upregulation/accumulation of various components of the amyloidogenic processing pathway, such as APP and BACE1.

### USP25 promotes BACE1 deubiquitination and APP β cleavage.

**Figure 5:**
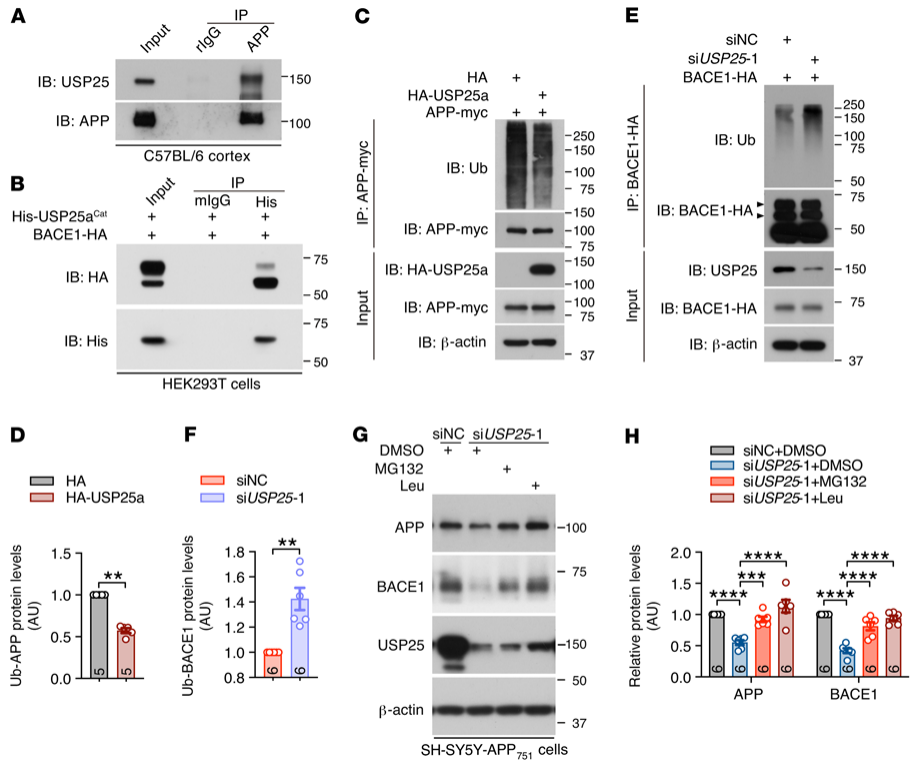
USP25 deubiquitinates and stabilizes APP and BACE1. (**A**) Co-IP of endogenous APP and USP25 in C57BL/6 mouse cortical lysates. Rabbit IgG was used as a negative control. (**B**) Co-IP of the purified His-tagged USP25a catalytic domain (His-USP25a^Cat^) and exogenously expressed BACE1-HA protein in HEK293T cells. Mouse IgG was used as a negative control. (**C** and **D**) Immunoblot analysis of polyubiquitinated APP-myc in HEK293T cells upon HA-USP25a overexpression. *n* = 5. (**E** and **F**) Immunoblot analysis of polyubiquitinated BACE1-HA in HEK293T cells upon *USP25* knockdown. *n* = 6. (**G** and **H**) Immunoblot analysis of APP and BACE1 in *USP25*-depleted SH-SY5Y-APP_751_ cell lysates treated with the proteasomal inhibitor MG132 (10 μM) or the lysosomal inhibitor leupeptin (Leu, 100 μg/mL). The intensity of each immunoblot band was normalized to that of the β-actin band. Values are shown in kDa in **A–C**, **E**, and **G**. *n* = 6. All data are presented as mean ± SEM. *P* values were determined by Student’s *t* test in **D** and **F** and by ordinary 1-way ANOVA with Dunnett’s post hoc analysis in **H**. **P < 0.01; ***P < 0.001; ****P < 0.0001.

To determine the underlying molecular mechanism by which USP25 regulates APP processing and Aβ generation, we further verified the interaction between USP25 and APP (Figure 5A and Supplemental Figure 4, A and B) and the interaction between USP25 and BACE1 but not BACE2 (Figure 5B and Supplemental Figure 4C) by coimmunoprecipitation (Co-IP). We found that USP25 overexpression reduced the amount of polyubiquitinated APP (Figure 5, C and D), and *USP25* knockdown increased polyubiquitinated BACE1 levels (Figure 5, E and F). Furthermore, we demonstrated that *USP25* depletion-mediated APP and BACE1 degradation can be reversed by inhibition of both the proteasomal and lysosomal degradation pathways (Figure 5, G and H, and Supplemental Figure 4, D and E).

**Figure 6:**
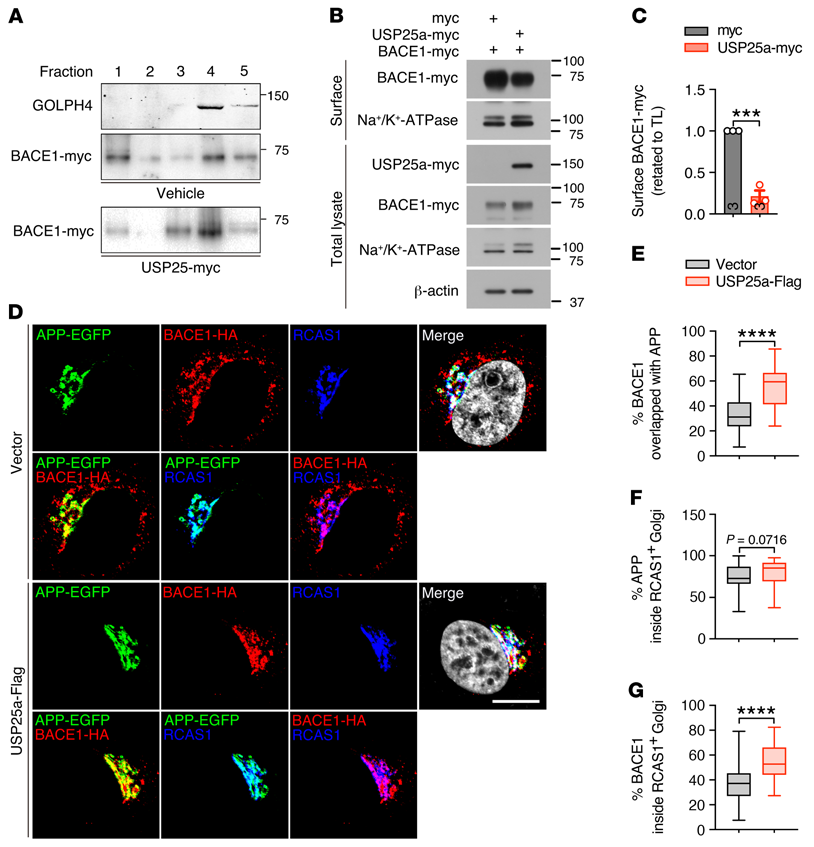
Overexpression of USP25 facilitates sorting of BACE1 into the Golgi apparatus. (**A**) Fractions from a 10%~50% sucrose density gradient were collected and concentrated by TCA precipitation and then subjected to immunoblot analysis. GOLPH4 was used as a Golgi marker. (**B** and **C**) Cell surface expression of BACE1-myc in HEK293 cells upon USP25a-myc overexpression was determined by surface biotinylation assay. Na^+^/K^+^ ATPase, a plasma membrane protein, was used as the internal control. Values are shown in kDa in **A** and **B**. TL, total lysate. *n* = 3 per group. (**D**–**G**) Immunofluorescence staining and colocalization analysis of APP-EGFP and BACE1-HA with RCAS1-positive Golgi in HeLa cells overexpressing USP25a-Flag or control vector. Scale bar: 10 μm. *n* = 43 (vector), *n* = 41 (USP25a-Flag). Data are presented as mean ± SEM (**C**) or median with minimum to maximum bars (**E**–**G**). *P* values were determined by Student’s *t* test in **C** and by the Mann-Whitney test in **E**–**G**. ***P < 0.001; ****P < 0.0001.

Furthermore, we performed sucrose gradient subcellular fractionation and found that USP25 overexpression increased the subcellular accumulation of BACE1 in the Golgi apparatus (Figure 6A). To further confirm the effect of USP25 on BACE1 trafficking, we performed a biotinylation assay to isolate the plasma membrane and found that USP25 overexpression markedly reduced the cell surface BACE1 distribution (Figure 6, B and C). Further immunostaining analyses revealed an increase in the sorting of BACE1 into the RCAS1-positive Golgi apparatus and colocalization of BACE1 with APP upon overexpression of USP25 (Figure 6, D–G). Together, our results identified APP and BACE1 as USP25 substrates and demonstrated that trafficking/processing of these components can be regulated by USP25-mediated deubiquitination.

### Pharmacological inhibition of USP25 ameliorates amyloid pathology in AD mice.

**Figure 7:**
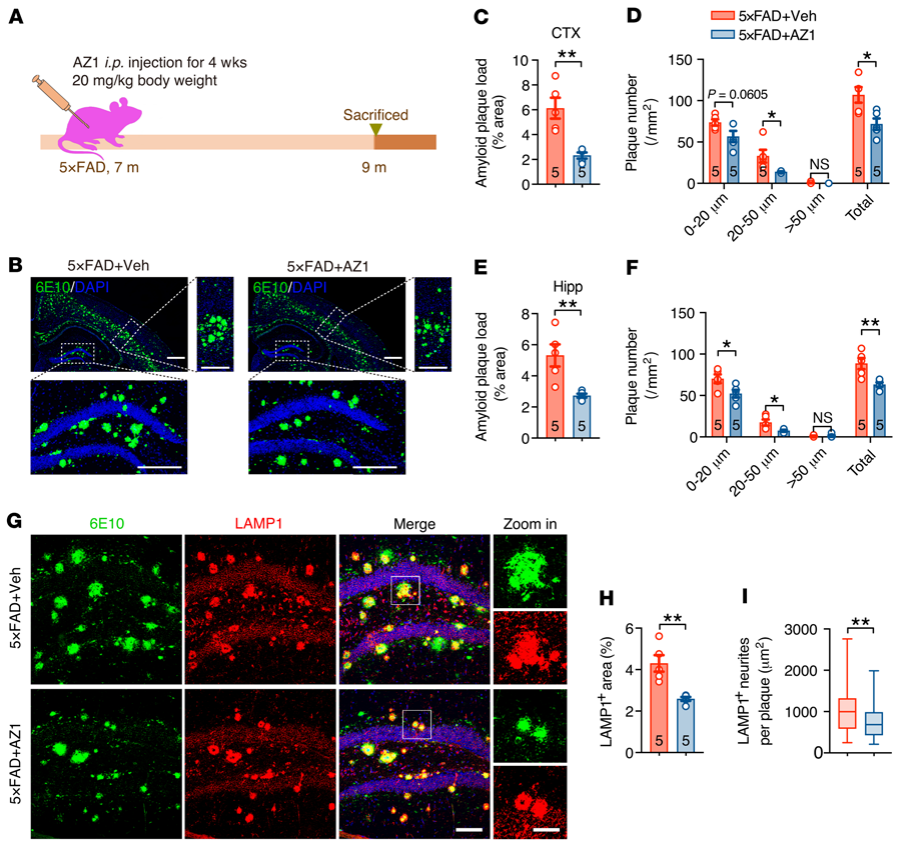
Pharmacological inhibition of USP25 ameliorates amyloid plaque deposition in 5×FAD mice. (**A**) Seven-month-old 5×FAD mice were intraperitoneally injected with vehicle or AZ1 (20 mg/kg/d) for 4 weeks and then subjected to pathological analyses. (**B**) Representative immunostaining of 6E10-positive amyloid plaques in 5×FAD+vehicle (Veh) and 5×FAD+AZ1 mice. Scale bar: 500 μm (merge); 250 μm (zoom in (high-magnification images to the right and below merge images)). (**C–F**) Quantification of 6E10-positive amyloid plaques in the mouse cortex (CTX) (**C** and **D**) and hippocampus (Hipp) (**E** and **F**). *n* = 5 mice per group. (**G**) Representative immunostaining of 6E10-positive amyloid plaques and LAMP1-positive dystrophic neurites in the hippocampi of 5×FAD+vehicle and 5×FAD+AZ1 mice. Scale bar: 100 μm (merge); 20 μm (Zoom in). (**H**) Quantification of LAMP1-positive dystrophic neurites in **G**. *n* = 5 mice per group. (**I**) Quantification of amyloid plaque–associated LAMP1-positive dystrophic neurites in **G**. *n* = 5 mice and 55 amyloid plaques (5×FAD+Veh), *n* = 5 mice and 47 amyloid plaques (5×FAD+AZ1). Data are presented as mean ± SEM (**C**–**F** and **H**) or median with minimum to maximum bars (**I**). *P* values were determined by Student’s *t* test in **C**–**F** and by the Mann-Whitney test in **H**, **I**, **K**, and **L**. *P < 0.05; **P < 0.01.

We took advantage of an in vitro enzymatic activity assay using a purified recombinant USP25 catalytic domain with a fluorogenic substrate consisting of ubiquitin derived at the C-terminus with rhodamine-110 (20) and found that compound AZ1 exhibited potent inhibitory activity against the USP25 enzyme with an IC_50_ value of 4.834 μM (Supplemental Figure 5A). Further immunoblot analyses confirmed that AZ1 administration reduced the protein amounts of APP and APP-CTFs in the cortices of 5×FAD mice compared with the vehicle controls (Supplemental Figure 5, B–E). To evaluate the effects of USP25 inhibition on amyloid deposition in vivo, we intraperitoneally injected 5×FAD mice with the USP25 inhibitor AZ1 (20 mg/kg/d, daily injection, 28 days) (Figure 7A). We subsequently performed histological analysis and found a reduction in the number of amyloid plaques in brain sections from AZ1-injected 5×FAD mice compared with those from 5×FAD mice administered solvent (Figure 7, B–F). Consistent with the reduction in amyloid plaques, AZ1 administration also resulted in a decrease in both LAMP1- and ATG9A-positive dystrophic neurites (Figure 7, G–I, and Supplemental Figure 5, F–H). These results indicated that pharmacological inhibition of USP25 ameliorated amyloid burden in the AD mouse model.

### USP25 expression correlates with AD pathology in the human AD brain.

**Figure 8:**
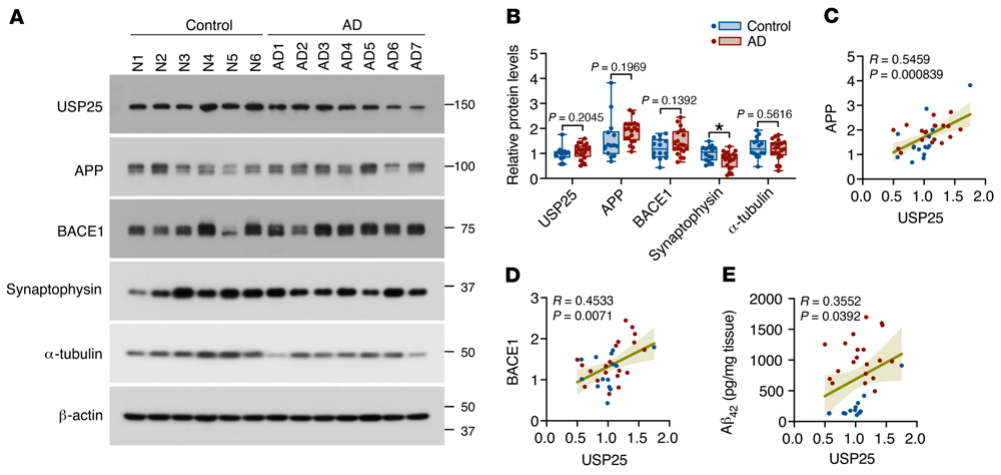
USP25 expression is correlated with APP and Aβ levels in brains from patients with AD. (**A**) Immunoblot analysis of USP25 and APP-processing-related proteins in the cortices of age-matched controls (normal) and patients with AD. Values are shown in kDa in **A**. Data are presented as median with minimum to maximum bars. (**B**) Quantification of the USP25, APP, BACE1, and synaptophysin protein amounts in **A**. *n* = 14 (control), *n* = 20 (AD). All data are presented as mean ± SEM. *P* values were determined by Student’s *t* test. *P < 0.05. (**C**–**E**) Correlation between USP25 expression and APP, BACE1, and Aβ_42_ levels. *n* = 34 (14 controls and 20 AD cases). *P* values were determined by Spearman’s rank correlation.

We examined USP25 expression in postmortem brain samples from individuals with AD and age-matched controls and found that USP25 protein levels remained unchanged in patients with sporadic AD compared with controls without AD; here, the synaptic marker synaptophysin was used as a control (Figure 8, A and B). However, we further performed Spearman’s correlation analysis and found a positive correlation between the amounts of USP25 and APP/BACE1/Aβ_42_ in the cortices of patients with AD (Figure 8, C–E), suggesting a potential role for the USP25-mediated amyloidogenic pathway in AD pathogenesis.

## Discussion

**Figure 9:**
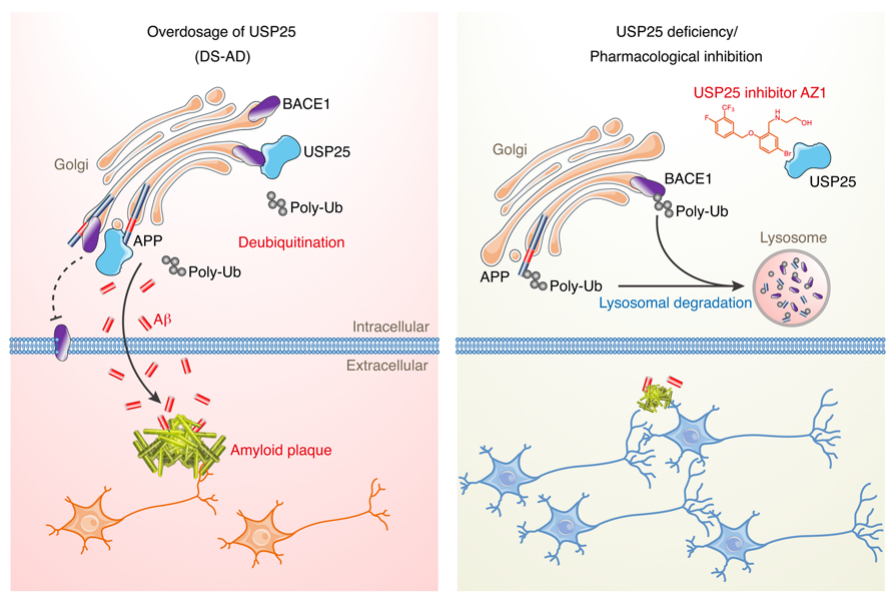
Targeting USP25 ameliorates amyloid pathology in Alzheimer’s brains. USP25 interacts with and deubiquitinates APP and BACE1 in the Golgi apparatus, resulting in amyloid pathology. Genetic depletion or pharmacological inhibition of USP25 induces lysosomal degradation of APP and BACE1 and reverses amyloid pathology in Alzheimer’s brains.

Because it is a major protein degradation pathway in all cell types, dysfunction of the ubiquitin-proteasome system results in various neurodegenerative diseases, including AD, DS, Parkinson’s disease, and Huntington’s disease (21, 22). Here, we confirmed that triplication of chromosome 21 genes aggravated AD-related amyloid pathology in a DS-AD mouse model. We found that, in addition to perturbing microglial homeostasis, USP25 plays a role in amyloid deposition by regulating APP processing and Aβ generation. Moreover, pharmacological inhibition of USP25 reversed amyloid pathology in 5×FAD mice, suggesting that USP25 inhibition could be an effective strategy for alleviating amyloid deposition in the AD brain (Figure 9).

A previous study demonstrated that trisomy 21 enhances Aβ plaque deposition independently of an extra copy of *APP* in a combined murine DS-AD model (J20×Tc1; ref. 4). In addition to APP, other genes and noncoding RNAs on chromosome 21 could also contribute to DS-AD neuropathology, such as *BACE2* (23), *CTSB* (24), *DYRK1A* (25), *ETS2* (26), *RCAN1* (27), *SUMO3* (28), and miR-155 (29). Our findings confirmed the pivotal role of USP25 in the regulation of Aβ generation and APP processing. The C-terminal fragment of APP contains 5 lysine residues (Lys 724, Lys 725, Lys 726, Lys 751, and Lys 763) that are potentially ubiquitinated (30, 31). E3 ligase FBL2-mediated ubiquitination of APP at Lys 726 resulted in APP degradation and reduced Aβ generation (32). Ubiquitination at APP Lys 763 sequesters APP in the Golgi apparatus and inhibits the maturation and proteolytic processing of APP (33). Moreover, ubiquitination of BACE1 at the C-terminus of Lys 501 led to the accumulation of BACE1 in late endosomes/lysosomes and thereby lysosomal degradation (34, 35). Here, we identified that USP25 deubiquitinates and prevents the lysosomal degradation of APP and BACE1. Moreover, USP25 facilitates the sorting of BACE1 into the Golgi apparatus. BACE1 is predominantly localized in endosomes and the late Golgi/trans-Golgi network (late Golgi/TGN); the low pH of endosomes and late Golgi/TGN allows for optimal cleavage activity of β secretase. Therefore, BACE1 trafficking into the Golgi apparatus enhances β cleavage of APP and Aβ production.

We previously found that the overdosage of USP25 induced microglia-mediated synapse elimination and neuroinflammation by deubiquitinating ATP6V0C and WDFY1 in the DS-AD brain (18). In the current study, we identified a role for neuronal USP25 in regulating APP processing and Aβ generation. Because USP25 can affect AD pathogenesis by influencing multiple cell types in the central nervous system, future studies characterizing the effects of conditional *Usp25* deletion in neurons are required to determine the cell type–specific effects of USP25 on amyloid deposition and other AD-related neurological phenotypes.

Although USP25 protein levels remain unchanged in patients with AD compared with controls without AD, more attention should be given to the catalytic activity of USP25, a ubiquitin-specific protease, and future studies will carefully characterize the regulatory mechanism of USP25 catalytic activity in the brain in the context of AD and related disorders. Moreover, to determine the genetic association between USP25 and AD pathogenesis, future genetic studies should identify key single nucleotide polymorphisms in the *USP25* gene in patients with AD.

In summary, our findings revealed that DS-related USP25 plays a role in AD-related amyloid pathology by modulating APP processing and degradation, providing a potential pharmacologic target for treating AD, especially in patients with DS.

## Methods

### Mouse strains.

BAC-Tg-USP25–transgenic mice and *Usp25*^−/−^ mice were generated as previously described (17, 18). Dp(16)1Yey/+ DS model mice (36, 37) (referred to as Dp16 mice in this study; stock no. 013530) and 5×FAD AD model mice (38) (stock no. 34840-JAX) were obtained from The Jackson Laboratory. Age-matched littermate male mice were used in this study.

### Human brain specimens.

Human AD brain specimens were obtained from the National Human Brain Bank for Health and Disease and the Brain Bank of University of Science and Technology of China. For specimen information, see Supplemental Table 1.

### DNA constructs and RNA interference.

HA-tagged USP25a and APP were constructed by using pCMV-HA as a backbone, HA-tagged BACE1 was constructed by using pCDNA3.1-HA/His as a backbone, myc-tagged USP25a and APP were constructed by using pCDN3.1-myc/His as a backbone, myc-tagged BACE1 was constructed by using pCDNA4-myc/His as a backbone, Flag-tagged BACE2 and USP25a were constructed by using pCDNA3.1-3×Flag as a backbone, and myc-tagged ubiquitin expressing vector pCW7 was constructed by using pRBG4-myc/His as a backbone.

For prokaryotic expression, the human USP25a catalytic domain (amino acids 157–706) was tagged with a His-tag and subcloned into the pET-His vector. The His-fused USP25a catalytic domain was expressed in *E*. *coli* BL21(DE3) cells and purified using Ni-NTA agarose (Qiagen, 1018244) as previously described (39).

Human *USP25* siRNA and control siRNA (siN0000001-1-5) were obtained from RiboBio, and the target sequences for *USP25* siRNA were as follows: si*USP25*-1, 5′-GTGAGCGATTTGCCCGAAT-3′; si*USP25*-2, 5′-GCATCAGGATTATAGGAAA-3′.

### Cell culture and transfection.

The APP_751_-overexpressing human neuroblastoma cell line SH-SY5Y-APP_751_ was maintained in DMEM (Thermo Fisher Scientific, C11995500BT) supplemented with 10% FBS (Thermo Fisher Scientific, 10270-106) and 200 μg/mL G418 (Thermo Fisher Scientific, 10131027). HEK293T (CBTCCCAS, SCSP-502) and HeLa (CBTCCCAS, SCSP-504) cells were cultured in DMEM (Thermo Fisher Scientific, C11995500BT) supplemented with 10% FBS (Thermo Fisher Scientific, 10270-106). DNA constructs and siRNA were transfected into cells using TurboFect Transfection Reagent (Thermo Fisher Scientific, R0534) and Lipofectamine RNAiMAX Transfection Reagent (Thermo Fisher Scientific, 13778100), respectively.

### Aβ_42/40_ ELISA.

The levels of Aβ_42_ and Aβ_40_ were measured by ELISA using the Human Aβ_42_ ELISA Kit (Thermo Fisher Scientific, KHB3441) and the Human Aβ_40_ ELISA Kit (Thermo Fisher Scientific, KHB3481), respectively, according to the manufacturer’s protocol.

### Immunoblot.

Immunoblot analyses were performed as previously described (18). The following antibodies were used: anti-APP (Millipore, MAB348, 1:1000), anti–APP-CTFs (Abcam, ab32136, 1:1000), anti–amyloid β (6E10) (against the N-terminus of human Aβ; BioLegend, 803001, 1:1000), anti-sAPPβ (BioLegend, 813401, 1:1000), anti–PS1-CTF (Sigma-Aldrich, P5110, 1:500), anti-USP25 (Abcam, ab187156, 1:1000), anti-ubiquitin (Santa Cruz Biotechnology, sc-8017, 1:500), anti-GOLPH4 (Abcam, ab57271, 1:1000), anti-Na^+^/K^+^ ATPase (Santa Cruz Biotechnology, sc-21712, 1:500), anti-myc (Thermo Fisher Scientific, 132500, 1:1000), anti-HA (Sigma-Aldrich, H6908, 1:1000), anti-Flag (Sigma-Aldrich, F7425, 1:1000), and HRP-conjugated secondary antibodies (Thermo Fisher Scientific, 31430 or 31460; 1:3,000). A previously described anti-PS1-NTF rabbit polyclonal antibody AB14 (1:1000; ref. 40) and a previously described anti-BACE1 mouse monoclonal antibody 3D5 (1:1000; ref. 41) were used.

### Co-IP.

Co-IP was performed as previously described (18). The following antibodies were used: anti-HA (Sigma-Aldrich, H6908, 1:200), anti-His (Proteintech, 66005-1-Ig, 1:200), isotype rabbit IgG (Santa Cruz Biotechnology, sc-3888, 1:200), and isotype mouse IgG (Santa Cruz Biotechnology, sc-2025, 1:200). A previously described anti-APP rabbit polyclonal antibody RU-369 (1:200; ref. 42) was used.

### Subcellular fractionation.

Cells were harvested in 1 mL PBS, centrifuged at 1000*g* for 60 seconds and resuspended in homogenization buffer (10 mM HEPES, pH 7.4, 1 mM EDTA, 0.25 M sucrose [8.56%], and protease inhibitor cocktail). The collected cells were homogenized with 15 strokes of a Kontes Dounce homogenizer and centrifuged at 1000*g* for 15 minutes to produce a postnuclear supernatant (PNS). PNS was loaded on top of a decreasing sucrose gradient consisting of 0.8 mL 2 M, 1.2 mL 1.3 M, 1.2 mL 1.0 M, and 0.8 mL 0.5 M sucrose in HB. Tubes were balanced and spun at 280,000*g* for 2 hours at 4°C. Nuclei and intact cells were precipitated in pellets by low-speed centrifugation at 1000*g* for 10 minutes. Samples were manually collected from the top of the tube, concentrated by TCA/acetone protein precipitation, and then subjected to immunoblot analysis.

### Cell surface biotinylation assay.

Cells were washed with PBS 3 times, and 1 mL of 1 mg/mL EZ-Link Sulfo-NHS-LC-Biotin (Thermo Fisher Scientific, 211335) in PBS was added and incubated with shaking at 4°C for 30 minutes. The reaction was quenched by washing the cells 2 times using ice-cold PBS with 1 M glycine. Cells were washed once with PBS before lysis in 600 μL lysis buffer (1% NP-40, 150 mM NaCl, 10 mM Tris-HCl, pH 7.4, supplemented with protease inhibitor cocktail). A total of 200 μL prewashed streptavidin agarose (Thermo Fisher Scientific, 20349) was added to the supernatant. The mixture was incubated overnight at 4°C on an orbital shaker. The resin was washed 3 times in PBS and boiled for 10 minutes in 2× SDS loading buffer to elute biotin-labeled protein. The eluted supernatant was subjected to immunoblot analysis.

### Immunocytochemistry.

Immunocytochemistry was performed as previously described (18). Transfected HeLa cells were subjected to immunostaining using the following antibodies and reagents: anti-RCAS1 (Cell Signaling Technology, 12290, 1:50), anti-HA (Abmart, M20003M, 1:1000), Alexa Fluor 568– and Alexa Fluor 635–conjugated secondary antibodies (Thermo Fisher Scientific, A-11031 and A-31577; 1:500), and DAPI (Sigma-Aldrich, D95542; 1 μg/mL). Confocal images were acquired with a ZEISS LSM 900 confocal microscope, and Manders’ colocalization coefficients were generated by ImageJ (NIH) with the “JACoP” tool.

### Immunohistochemistry.

Immunohistochemistry was performed as previously described (18). The following antibodies and reagents were used: anti–amyloid β {6E10) (BioLegend, 803001, 1:400), anti-LAMP1 (Abcam, ab24170, 1:200), anti-ATG9A (Abcam, ab108338, 1:200), Alexa Fluor 488– and Alexa Fluor 594–conjugated secondary antibodies (Thermo Fisher Scientific, A-11001 and A-11005; 1:500), thioflavin S (Sigma-Aldrich, T1892, 8 mM), and DAPI (Sigma-Aldrich, D95542; 1 μg/mL).

### Golgi staining.

Golgi staining was performed as previously described (18) using the FD Rapid Golgi Stain Kit (FD Neuro Technologies, PK401).

### Pharmacological treatment.

Leupeptin (MCE, HY-18234A, 100 μg/mL and ammonium chloride (Sigma-Aldrich, A9434, 50 mM) were used to inhibit lysosomal protein degradation. MG132 (MCE, HY-13259, 10 μM) was used to inhibit proteasomal protein degradation.

### In vitro deubiquitination assays using ubiquitin-rhodamine 110.

AZ1 activity was analyzed against the purified His-fused USP25 catalytic domain (His-USP25a^Cat^, amino acids 157–706) using ubiquitin-rhodamine 110 (R&D System, U-555-050) as a substrate in 384-well white plates (Corning, 3570) as previously described (20). Briefly, different dosages of AZ1 (TubePharm; AZ1 was dissolved in DMSO, and the final concentration of DMSO was 0.1%), from 0 to 100 μM (final concentration), were incubated with 33.3 nM His-USP25a^Cat^ at room temperature for 20 minutes in assay buffer containing 50 mM HEPES, pH 7.4, 0.5 mM EDTA, 1 mM tris-(2-carboxyethyl) phosphine (TCEP), and 1 mg/mL bovine serum albumin. After incubation with 133.3 nM ubiquitin-rhodamine at room temperature for 30 minutes, 25 mM citric acid was added to stop the reaction. Spectrofluorometry readings were acquired using a Spark Multimode Microplate Reader (Tecan).

### Administration of AZ1.

AZ1 (TubePharm) was dissolved in 5% DMSO and +95% corn oil as previously described (18). Seven-month-old male 5×FAD mice and littermate WT mice were intraperitoneally injected with AZ1 at a dose of 20 mg/kg for 4 weeks, whereas the control group was administered an equivalent amount of vehicle solvent.

### Open field test.

The open field test was conducted in a home cage. The experimental mice were allowed to freely explore for 10 minutes, and the total distance and percentage of time the mice spent in the center zone were calculated automatically using Smart Video Tracking Software 3.0 (Panlab, Harvard Apparatus).

### MWM test.

The MWM test was performed as previously described (18). Briefly, the MWM test was performed in a circular tank filled with opaque water, and 4 reference cued shapes were affixed to the walls surrounding the tank. In the hidden platform training phase, the mice were placed into the maze at 1 of 4 random points and allowed to search for the hidden platform for 60 seconds. If a mouse failed to find the platform within 60 seconds, it was guided to the platform and allowed to rest for 10 seconds. The time taken to find the hidden platform was recorded by Smart Video Tracking Software 3.0 (Panlab, Harvard Apparatus). In the platform test phase, the hidden platform was removed. The time spent in each quadrant and the number of crossings over the platform were scored.

### FC test.

The FC test was conducted in the fear conditioning chamber (Panlab, Harvard Apparatus) and performed as previously described (18). Briefly, on the training day, the mice were allowed to explore the chamber for 2 minutes, followed by a white noise stimulus (30 s, 60 dB), which served as a conditioned stimulus, and a 0.5 mA foot shock, which presented as an unconditioned stimulus during the last 2 seconds of white noise. A 1-minute interval separated the 3 conditioning trials. The contextual test was conducted on day 2; the mice were allowed to explore the same conditioning chamber for 5 minutes, and freezing behaviors were scored.

### Statistics.

All the data were analyzed using GraphPad Prism software (version 8). Detailed statistical methods are provided in each figure legend and include 1-way ANOVA with Tukey’s post hoc analysis, 1-way ANOVA with Dunnett’s post hoc analysis, Spearman’s rank correlation, Mann-Whitney test, and 2-tailed Student’s t tests. All data are presented as mean ± SEM or as median with minimum to maximum bars.

### Study approval.

All animal experiments were approved by the Institutional Animal Care and Use Committee of Xiamen University. Human studies were approved with informed consent by the ethical review board at the School of Medicine, Xiamen University (project no. XDYX2021-21).

## Author contributions

WS and Xin Wang conceived the study and designed the experiments; Q Zheng, BS, GL, FC, MW, Yingjun Zhao, LJ, TG, MS, HH, Yini Zhao, AD, LZ, FZ, ZZ and TYH performed the experiments; Q Zheng, BS, GL, FC, Ying Zhou, XFZ, HL, XZ, HZ, CD, HQ, Yun Zhang, Q Zhang, Xu Wang, YW, HX, Xin Wang, and WS analyzed and contributed reagents/materials/analysis tools; Q Zheng, BS, FC, Xin Wang, and WS wrote the paper. All authors reviewed the manuscript.

## Supplementary Material

Supplemental data

## Version 1 03/01/2022

Electronic publication
