# Supplementary material for: USP25 inhibition ameliorates Alzheimer’s pathology through the regulation of APP processing and Aβ generation
Source: J Clin Invest. 2022 Mar 1;132(5):e152170. doi: 10.1172/JCI152170 (PMC8884900; doi:10.1172/JCI152170)
Supplement: Supplemental data [file jci-132-152170-s106.pdf]

Supplemental Material

USP25 inhibition ameliorates Alzheimer's pathology through regulating APP processing and Aβ generation

Qiuyang Zheng, Beibei Song, Guilin Li, Fang Cai, Meiling Wu, Yingjun Zhao, Lulin Jiang, Tiantian Guo, Mingyu Shen, Huan Hou, Ying Zhou, Yini Zhao, Anjie Di, Lishan Zhang, Fanwei Zeng, Xiu-Fang Zhang, Hong Luo, Xian Zhang, Hongfeng Zhang, Zhiping Zeng, Timothy Huang, Chen Dong, Hong Qing, Yun Zhang, Qing Zhang, Xu Wang, Yili Wu, Huaxi Xu, Weihong Song, and Xin Wang

Supplemental Figures

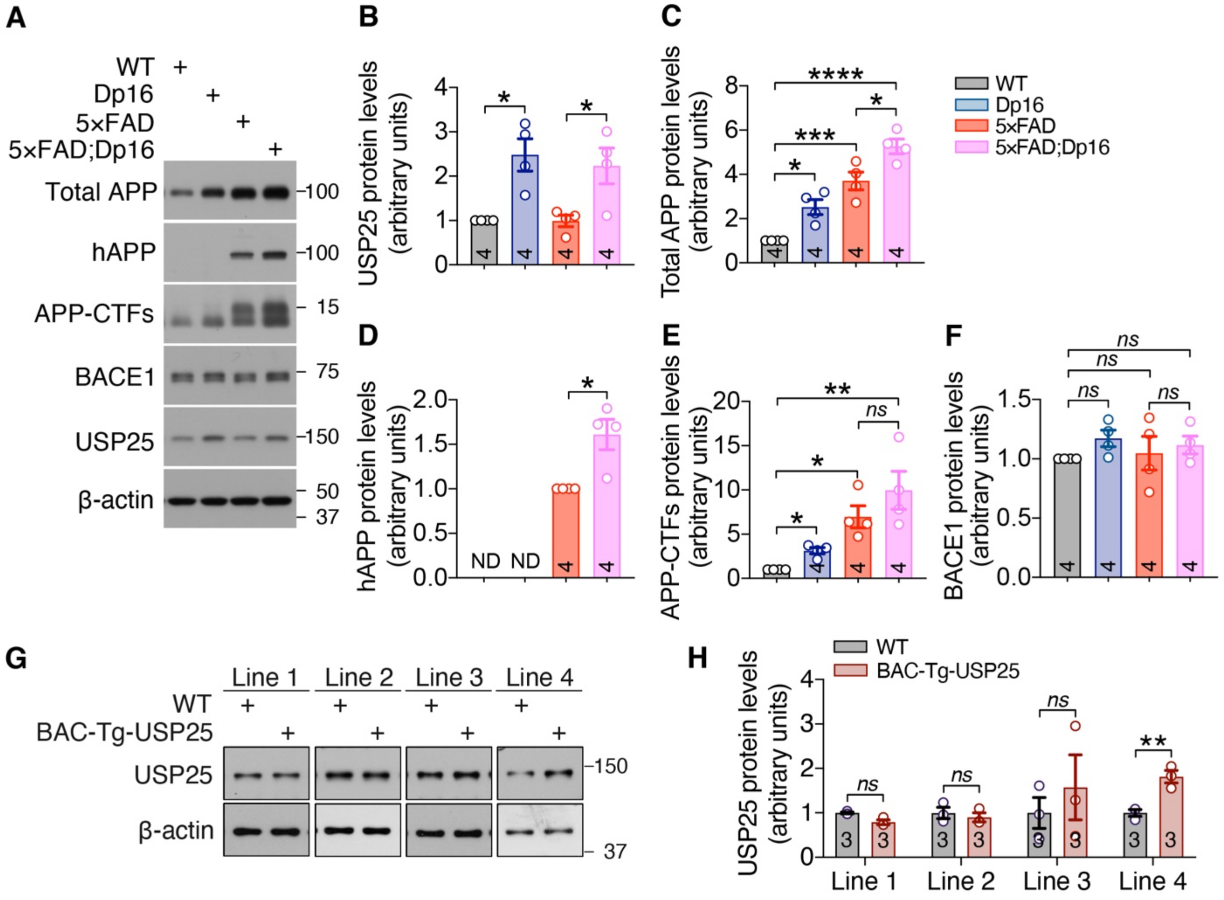

Supplemental Figure 1. Trisomy 21 aggravates Alzheimer's pathology. (**A-F**) Immunoblot analysis of APP processing-related proteins in the cortex of 5-month-old WT, Dp16, 5×FAD, and 5×FAD;Dp16 mice. ND, not detected. *n* = 4 mice per group. (**G** and **H**) Immunoblot analysis of USP25 in the hippocampi of 2-month-old BAC-Tg-USP25 mice in lines 1-4. *n* = 3 mice per group. All data are presented as mean ± SEM. *P* values were determined by ordinary one-way ANOVA with Tukey's *post hoc* analysis in (B, C, E and F) and by Student's *t* test in (D and H). *ns*, not significant; **P*<0.05; ***P*<0.01; ****P*<0.001; *****P*<0.0001.

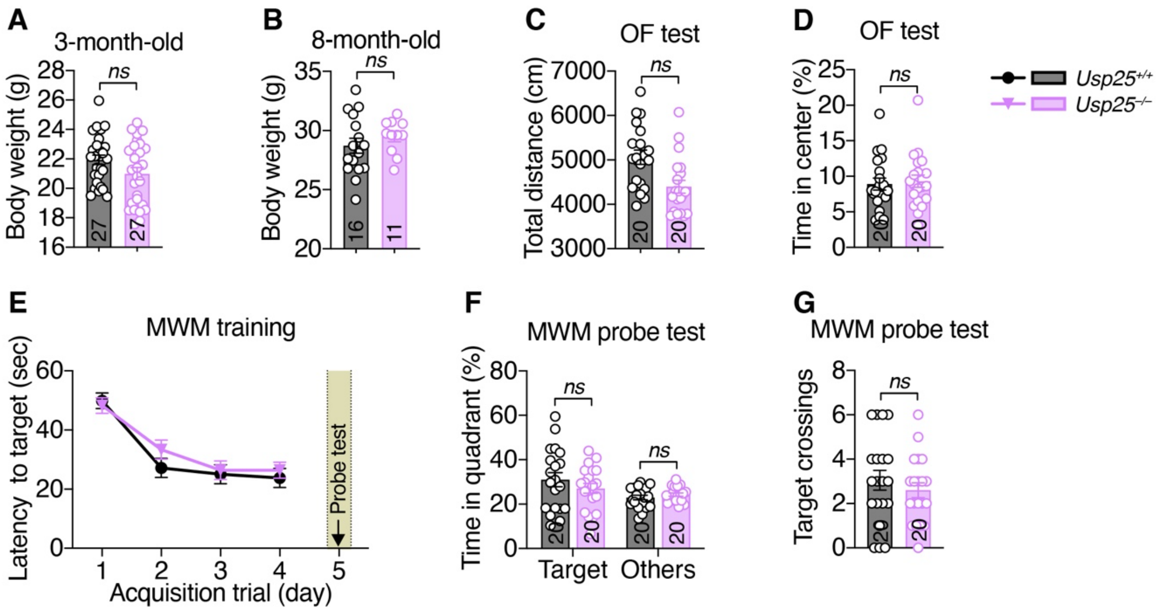

Supplemental Figure 2. ***Usp25*** **knockout mice are grossly healthy.** (**A** and **B**) The body weights of 3-month-old (**A**) and 8-month-old (**B**) *Usp25*$^{+/+}$ and *Usp25*$^{+/-}$ mice. n = 11 to 27 mice per group. (**C-G**) Behavioral test of 3-month-old *Usp25*$^{+/+}$ and *Usp25*$^{+/-}$ mice. (**C** and **D**) Total distance traveled (**C**) and percentage of time the mice spent (**D**) in the open field (OF) test. n = 20 mice per group. (**E**) MWM test results depicting escape latency, defined as the time taken to find a hidden platform in the 4-day training phase. (**F** and **G**) MWM probe test results. The percentage of time spent in the quadrants (**F**) and the number of crossings over the platform (**G**) were quantified. n = 20 mice per group. All data are presented as mean ± SEM. P values were determined by Student's t test in (A-D, F and G). *ns*, not significant.

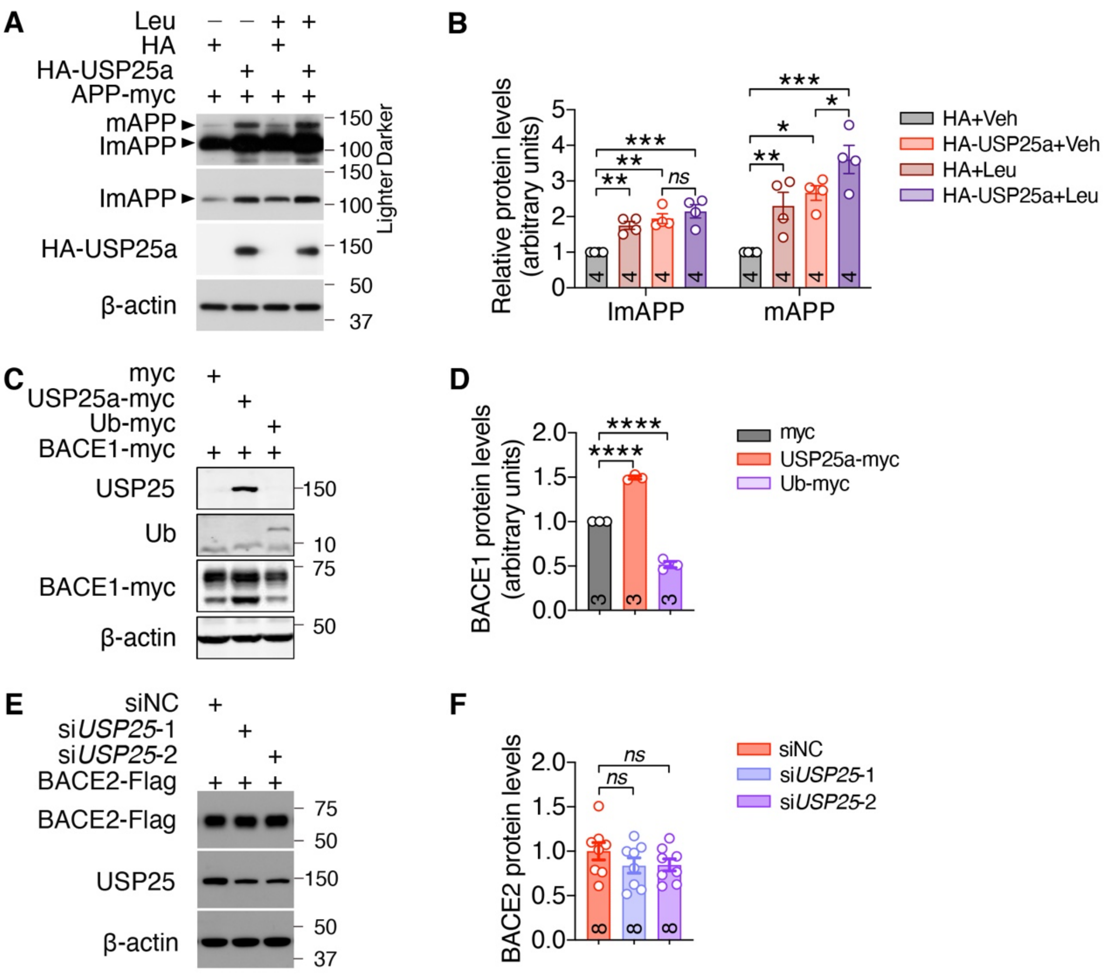

Supplemental Figure 3. Overexpression of USP25 increases the expression of APP and BACE1 in vitro. (**A**) Immunoblot analysis of APP protein amounts in APP-overexpressing HEK293T cells upon HA-USP25a transfection. Cells were treated with either leupeptin (Leu, +) or vehicle (–) for 6 h. (**B**) Quantification of (A), immature APP (ImAPP) and mature APP (mAPP) proteins. *n* = 4. (**C**) Immunoblot analysis of BACE1-myc expression in HEK293 cells upon USP25a-myc or ubiquitin (Ub)-myc overexpression. (**D**) Quantification of (C). *n* = 3. (**E**) Immunoblot analysis of exogenously expressed BACE2 in HEK293T cells upon *USP25* siRNA treatment. (**F**) Quantification of (E). *n* = 8. All data are presented as mean ± SEM. *P* values were determined by ordinary one-way ANOVA with Tukey's *post hoc* analysis in (B), by ordinary one-way ANOVA with Dunnett's *post hoc* analysis in (D), and by ordinary one-way ANOVA with Kruskal-Wallis *post hoc* analysis in (F). *ns*, not significant; *P<0.05; **P<0.01; ***P<0.001; ****P<0.0001.

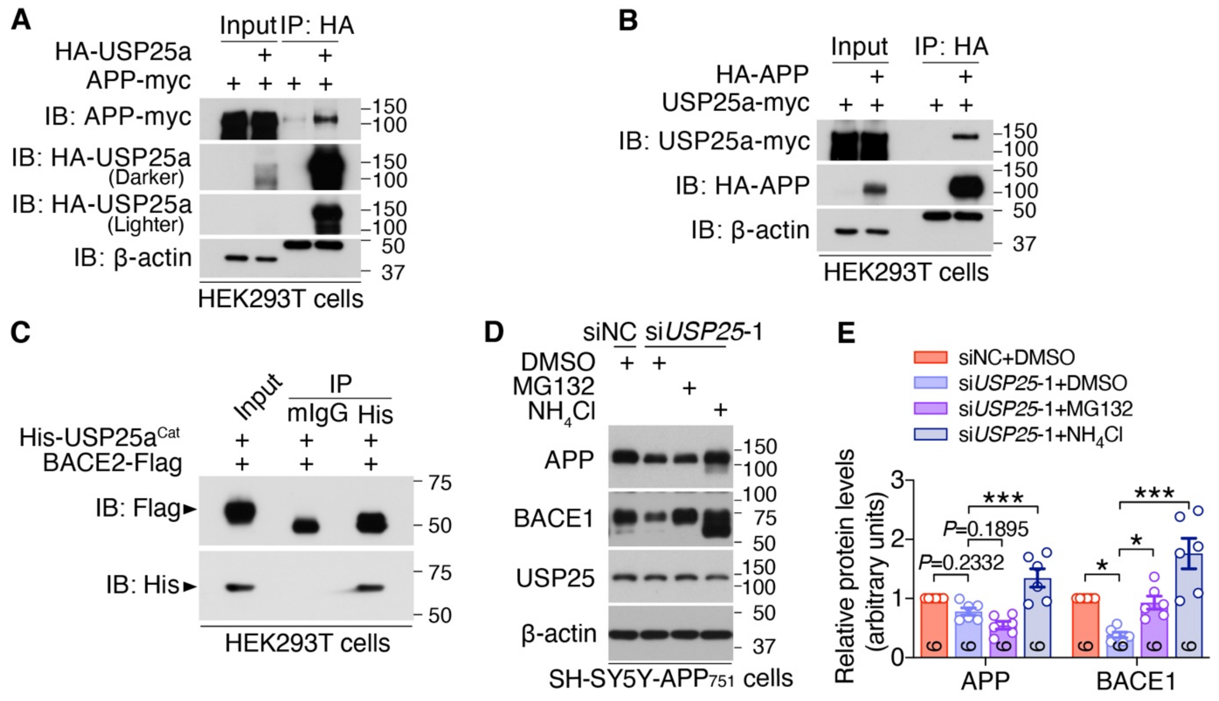

Supplemental Figure 4. USP25 interacts with and stabilizes APP and BACE1. (**A**) Co-IP of exogenously expressed HA-USP25a and APP-myc proteins in HEK293T cells. (**B**) Co-IP of exogenously expressed HA-APP and USP25a-myc proteins in HEK293T cells. (**C**) Co-IP of the purified His-tagged USP25a catalytic domain (His-USP25a^{Cat}) and exogenously expressed BACE2-Flag in HEK293T cells. (**D** and **E**) Immunoblot analysis of APP and BACE1 in *USP25*-depleted SH-SY5Y-APP_{751} cells treated with the proteasomal inhibitor MG132 (10 μM) or the lysosomal inhibitor NH_4Cl (50 mM). The intensity of each immunoblot band was normalized to that of the β-actin band. $n = 6$. All data are presented as mean ± SEM. *P* values were determined by ordinary one-way ANOVA with Dunnett's *post hoc* analysis in (E). *$P<0.05$; ***$P<0.001$.

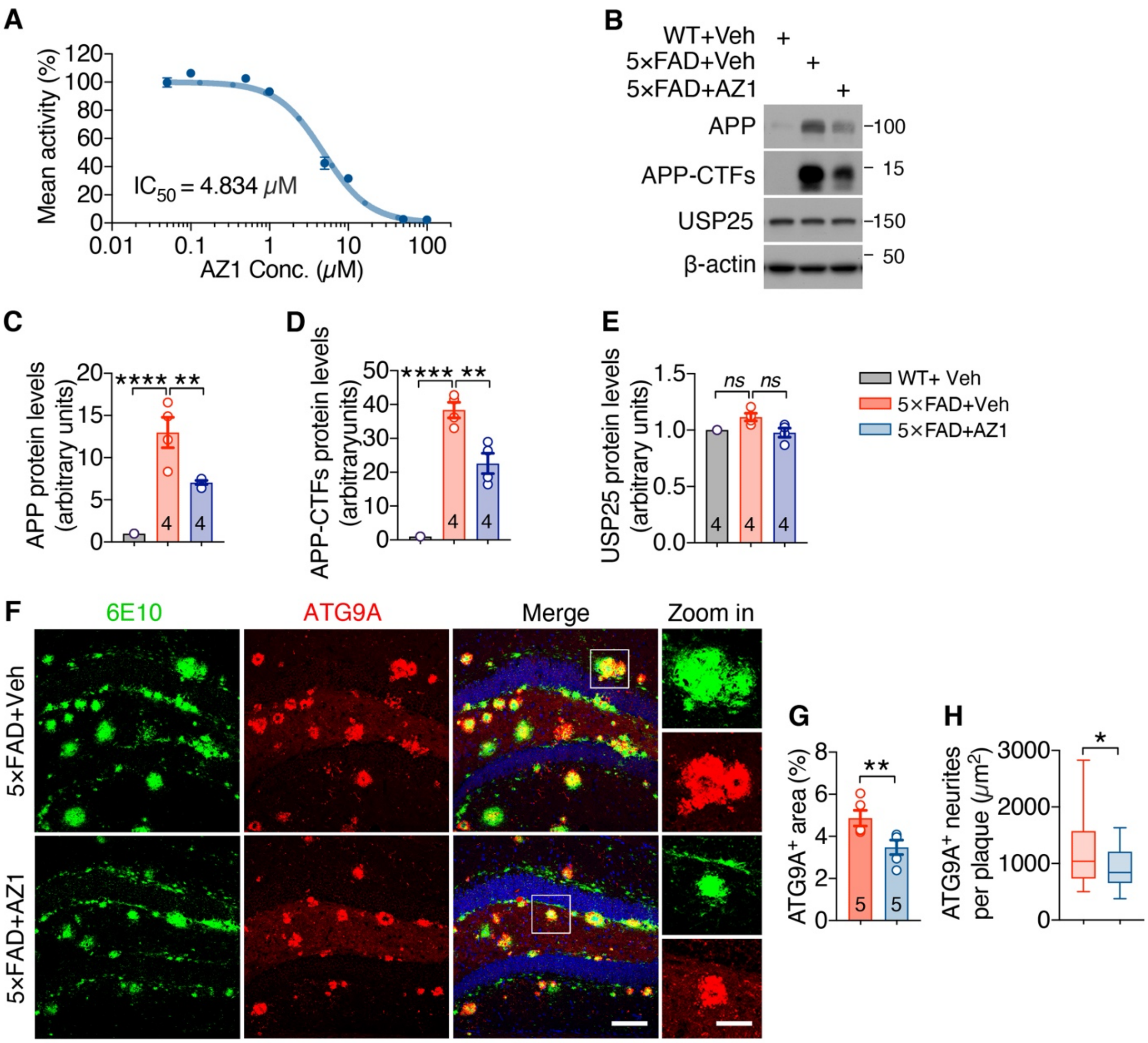

Supplemental Figure 5. AZ1 inhibits the deubiquitinase activity of USP25. (**A**) Dose response profiling of AZ1 against the purified USP25 catalytic domain using ubiquitin-rhodamine 110 as a substrate. (**B**-**E**) Immunoblot analysis of APP and APP-CTFs in the cortex of WT+Vehicle (Veh), 5×FAD+Vehicle, and 5×FAD+AZ1 mice. *n* = 4 mice per group. (**F**) Representative immunostaining of 6E10-positive amyloid plaques and ATG9A-positive dystrophic neurites in the hippocampi of 5×FAD+vehicle and 5×FAD+AZ1 mice. Scale bar (merge), 100 µm; scale bar (zoom in), 20 µm. (**G**) Quantification of ATG9A-positive dystrophic neurites in (F). *n* = 5 mice per group. (**H**) Quantification of amyloid plaque-associated ATG9A-positive dystrophic neurites in (F). *n* (5×FAD+Veh) = 5 mice and 40 amyloid plaques, *n* (5×FAD+AZ1) = 5 mice and 40 amyloid plaques. Data are presented as mean ± SEM (A, C-E, and G) or median with minimum to maximum bars (H). *P* values were determined by ordinary one-way ANOVA with Dunnett's *post*

hoc analysis in (C-E) and by the Mann-Whitney test in (G and H). *ns*, not significant; *$P<0.05$; **$P<0.01$; ****$P<0.0001$.

Supplemental Tables

Supplemental Table 1. Information of the postmortem brain samples.

NO.	Case ID	Disorder	Age	Gender	Post mortem delay (min)
1	N1	Control	81	Male	260
2	N2	Control	75	Female	250
3	N3	Control	79	Male	230
4	N4	Control	95	Male	660
5	N5	Control	83	Male	1480
6	N6	Control	92	Male	301
7	N7	Control	84	Female	240
8	N8	Control	77	Female	1430
9	N9	Control	70	Female	200
10	N10	Control	69	Female	1657
11	N11	Control	75	Male	368
12	N12	Control	87	Male	475
13	N13	Control	51	Male	445
14	N14	Control	71	Female	525
15	AD1	AD	79	Male	300
16	AD2	AD	90	Female	230
17	AD3	AD	67	Male	307
18	AD4	AD	75	Male	210
19	AD5	AD	97	Female	390
20	AD6	AD	79	Male	310
21	AD7	AD	83	Female	1440
22	AD8	AD	81	Male	180
23	AD9	AD	70	Female	570
24	AD10	AD	93	Female	288
25	AD11	AD	83	Female	225
26	AD12	AD	86	Female	120
27	AD13	AD	99	Female	240
28	AD14	AD	83	Male	270
29	AD15	AD	85	Male	930
30	AD16	AD	91	Female	520
31	AD17	AD	71	Female	490
32	AD18	AD	73	Male	570
33	AD19	AD	91	Male	266
34	AD20	AD	88	Male	700
